# Annoyance, Sleep and Concentration Problems due to Combined Traffic Noise and the Benefit of Quiet Side 

**DOI:** 10.3390/ijerph120201612

**Published:** 2015-01-29

**Authors:** Theo Bodin, Jonas Björk, Jonas Ardö, Maria Albin

**Affiliations:** 1 Division of Occupational and Environmental Medicine, Lund University, SE-221 85 Lund, Sweden; E-Mails: jonas.bjork@med.lu.se (J.B.); maria.albin@med.lu.se (M.A.); 2 Department of Physical Geography and Ecosystem Science, Lund University, SE-223 62 Lund, Sweden; E-Mail: Jonas.ardo@nateko.lu.se

**Keywords:** noise, road traffic, railway, annoyance, sleep, cognition, quiet side

## Abstract

*Background*: Access to a quiet side in one’s dwelling is thought to compensate for higher noise levels at the most exposed façade. It has also been indicated that noise from combined traffic sources causes more noise annoyance than equal average levels from either road traffic or railway noise separately. *Methods*: 2612 persons in Malmö, Sweden, answered to a residential environment survey including questions on outdoor environment, noise sensitivity, noise annoyance, sleep quality and concentration problems. Road traffic and railway noise was modeled using Geographic Information System. *Results*: Access to a quiet side, *i.e.*, at least one window facing yard, water or green space, was associated with reduced risk of annoyance OR (95%CI) 0.47 (0.38–0.59), and concentration problems 0.76 (0.61–0.95). Bedroom window facing the same environment was associated to reduced risk of reporting of poor sleep quality 0.78 (0.64–1.00). Railway noise was associated with reduced risk of annoyance below 55 dB(A) but not at higher levels of exposure. *Conclusions*: Having a window facing a yard, water or green space was associated to a substantially reduced risk of noise annoyance and concentration problems. If this window was the bedroom window, sleeping problems were less likely.

## 1. Introduction

Road traffic noise is a growing health hazard in the urbanized world. Conservative estimates assume that at least one million healthy life years are lost every year from traffic related noise in the western part of Europe [1]. Although Sweden is a fairly quiet country compared to continental Europe, noise derived from aircraft, railway and road traffic has increased over the years and is predicted to increase by 23%–27% until 2020 compared to the levels of 2001 [2]. 

The main health burden related to road traffic noise stems from noise annoyance and sleep disturbance [1]. Traffic noise has also been linked with several other adverse effects on life quality and health, including increased risk of hypertension, myocardial infarction, and in some studies stroke and diabetes, although these outcomes are less well studied [3,4,5,6,7]. 

The societal costs related to road traffic noise are most likely very high. A recent study from Switzerland concluded that one night’s undisturbed sleep was worth CHF 7–24 (approx. 5–15 EUR) depending on severity of disturbance [8]. In the EU 22, the social cost of road traffic noise is estimated to be at least €38 (30–46) billion per year, which is approximately 0.4% of gross domestic product (GDP) and approximately one third of the social costs for traffic related accidents [9].

Disturbed sleep due to noise from aircraft, railway and road traffic has been shown in laboratory settings as well as in field studies [10,11]. Traffic noise affects several aspects of sleep quality. The time it takes to fall asleep is prolonged in relation to noise exposure. Increased heart rate, blood pressure and changes in electroencephalogram (EEG) during sleep have been recorded in relation to traffic noise exposure. Traffic noise also affects subjective sleep quality and is associated with the notion of not being totally rested after a whole night’s sleep. Awakenings during the night and premature awakening in the morning have been shown in short-term studies but it is concluded that substantial habituating effects exist [12]. However, habituation has not been observed with regard to arousal measured by increased heart rate or EEG-patterns [11,13].

In policy discussions and research, there is an increasing interest in the benefit of access to a quiet side of the dwelling [14]. There is hope that access to a side, sheltered from noise, would allow people to sleep with bedroom windows open at night or to make use of balconies or other outdoor spaces close to the residency, thus compensating for high noise levels at the most exposed façade. This beneficial effect has earlier been found on noise annoyance and sleep quality [15,16] but definitions have varied as well as the effect size and it is unclear whether the effect is sustained at higher noise levels. Also, there have been no reports on quiet side’s effect on cognition.

Noise from different traffic sources have different characteristics and have been shown to have different impact on sleep at equal nocturnal noise exposure levels. A review on this topic, with pooled data from 24 different studies, found that noise from aircraft was associated with more sleep disturbance than road traffic noise, which subsequently was found to be associated with more sleep disturbance than railway noise [17]. This pattern has resulted in so called “railway bonus”, often of 5 dB(A), which has been implemented in noise legislation in a number of European countries. In a Swedish study it was indicated that noise from combined exposure correlated to reporting more noise annoyance than equal average levels from either road traffic or railway noise separately [18] and in recent years this bonus has started to be questioned, especially at noise levels above 55 dB(A) [19,20]. 

### Aims and Hypothesis

The first aim of this study was to evaluate how, at different levels of exposure, access to a quiet side of one’s dwelling affected sleep, concentration and noise annoyance. The second aim was to investigate the impact of noise exposure from combined sources compared to being exposed dominantly to road traffic or railway noise. 

## 2. Materials and Methods 

### 2.1. Exposure Assessment 

In order to fulfill the EU directive regarding assessment and management of environmental noise [21], the city of Malmö contracted the consultant firm ÅF-Ingemansson AB to do an inventory and assessment of the environmental noise in Malmö [22].

Data used for the assessment included geometries of roads, buildings, elevation data, ground types, noise protection installations such as noise barriers, and railways. Road traffic included number of standard and heavy vehicles and their diurnal distribution. Railway traffic data included number and type of trains, train length and velocity (see [22] for details).

Calculations were performed according to the standard Nordic prediction model [23,24,25] for assessment of noise from road traffic and railway traffic, using SoundPLAN version 6.4 (Braunstein + Berndt GmbH, Backnang, Germany). 

Road traffic noise, and railway noise were modeled separately and combined. When comparing adverse effects of noise from different sources we applied a concept of dominant source, *i.e.*, if there was a difference of 3 dB(A) or more between railway and road traffic noise the source with higher levels was considered the dominant one.

Road traffic and railway noise were calculated for the most exposed façade of each building where the study participants had their home addresses. Information on which floor or where in the building each person lived was not available.

### 2.2. Study Population

This study, was based on 2612 answers to the survey “Undersökning om boendemiljö och hälsa” (“Survey regarding household environment and health”) which was sent to individuals aged 18–79 residing in Malmö on 12 April 2007, a city which at the time had a population of 207,781 within this age span. The selection of survey participants was made through a random sampling of 800 individuals from six different strata based on road traffic and railway noise exposure levels using a simplified version of the Nordic prediction model. [24,25] The six strata were based on three levels of road traffic noise (<40 dB(A); 40–60 dB(A) and >60 dB(A)) with and without simultaneous occurrence of railway noise. One extra strata consisting of an additional 800 individuals was added based on living nearby construction sites related to a major railway tunnel project (Citytunneln), but was not included in the present study. Among the 4800 individuals from the six strata above, 2612 (54%) chose to respond to the survey. Answers were collected, including two reminders, during the period June–August 2007. The study was conducted in accordance to Swedish law of ethics at the time, which did not require IRB approval for survey studies. 

### 2.3. Assessment of Adverse Effects

Adverse effects of noise exposure were assessed through self-reporting. The survey was, with some modifications, adapted from Öhrstrom *et al*. [26] and included a range of questions designed to assess (A) Housing and living conditions (e.g., type of dwelling, surrounding environment, satisfaction with area); (B) Annoyance due to environmental exposure (Noise from industries and construction sites, smell, fumes, vibrations and noise from neighbours); (C) Annoyance due to road and railway noise (including effects on everyday life such as radio and TV listening, conversations, sleep and rest); (D) Health conditions (hearing impairment, asthma, hypertension, mental health); (E) Sleep and rest; (F) Basic facts, work and education. 

Noise annoyance was assessed using a Swedish translation of a 5-point ISO/TS 15666 verbal scale for assessment of noise annoyance by means of social and socio-acoustic surveys [27]. *“During the last 12 months, how disturbed have you been because of traffic noise (total/rail/road/air traffic) at home?” (1 = “Not at all bothered or annoyed”, 2 = “Slightly bothered or annoyed”, 3 = “Moderately bothered or annoyed”, 4 = “Very bothered or annoyed”, and 5 = “Extremely bothered or annoyed”)*. Sleep and concentration problems were assessed through the following questions, unrelated to noise: *“How do you usually sleep?” (*5-point scale*) 1 = “Very poorly”, 2 = “Poorly”, 3 = “Not very good”, 4 = “Pretty good”, 5 = “Very good”*; *“Do you usually have difficulties concentrating on what you want to do?” (4-point scale) 1 = “Rarely/Never”, 2 = “A few times per month”, 3 = “A few times per week” and 4 = “Every day”.*

To be annoyed in this study is defined as stating to be *“Moderately annoyed”, “Very annoyed”,* or *“Extremely annoyed”* on the 5-point scale described above. Poor sleep quality was defined as *“Not very good”, “Poor” or “Very poor”* on the 5-point scale. Concentration problems were defined as *“A few times per week” or “Every day”* on a 4-point scale.

### 2.4. Assessment of Quiet Side

We assessed access to quiet side using both (1) indirect and (2) direct, self-assessment of quiet side in the survey: (1) Windows facing green space. *“Does your dwelling have windows facing directly towards…”, “Large street or road”, “small street”, “railway”, “industrial area or industry”, “a yard, garden, water or green space”, “something else…”* There was also a question with identical alternatives, asking specifically for bedroom window direction, *i.e.*, *“Does your bedroom window directly face...”.* For these questions, having a window facing green space (*“a yard, garden, water or green space”*) was considered as a proxy for having access to a quiet side in the dwelling; (2) Access to quiet indoor space. *Do you have access to a quiet indoor space in your dwelling where you are not disturbed by noise?” “Yes”/“No”*.

### 2.5. Statistical Analysis

We applied Spearman correlation analysis and logistic regression using SPSS Statistics 20.0.2 for Mac OSX in order to investigate associations between noise exposure and noise annoyance, sleep and concentration problems. In all analyses noise exposure has been defined as L_Aeq24h_ road, rail or both combined. Noise has been entered both as continuous and categorical variables (<40; 40–44; 45–49; 50–54; 55–59; ≥60 dB(A)). In all regression models, the reference category has been L_Aeq24h_ < 40 dB(A), also when noise has been entered as a continuous variable. Adjusted models included factors considered *a priori* as relevant in relation to noise annoyance, sleep and concentration problems or anticipated to confound associations with noise exposure, including age (continuous); sex (male/female); Body mass index (BMI continuous); educational level (university and high school *vs.* up to 9 years primary school); strained economy (having difficulties paying one’s bills more than half of the time the last 12 months *vs.* “*never*” and *“a few times”*); country of birth (Sweden *vs.* all others); civil status (married and co-living *vs.* single and divorced/widowed), smoking (current use *vs.* former use and never) and hearing impairment (*“yes”/”no”*). Age was also entered as a categorized variable with no changes in results, as shown in Table 1. Effect estimates were presented as odds ratios (ORs) with 95% confidence intervals (CIs). Interaction between windows facing a green space and noise exposure was investigated by adding a multiplicative interactive term (*a* × *b*) based on road traffic noise exposure (*a*; 5 dB(A) intervals) and windows facing a green space (*b*; categorical, yes/no). *p*-values below 0.05 were regarded as statistically significant. 

**Table 1:** Responders and non-responders by sex, age and noise exposure using the simplified Nordic prediction model.

		Non-Responders	Responders	Response Rate
		n	n	%
Total		2188	2612	54%
Sex				
	Women	979 (45%)	1420 (54%)	59%
	Men	1209 (55%)	1192 (46%)	50%
Age				
	18–29	736 (34%)	484 (19%)	40%
	30–49	883 (40%)	971 (37%)	52%
	50–64	387 (18%)	694 (27%)	64%
	65–79	182 (8%)	463 (18%)	72%
Railway L_Aeq24h_ dB(A)				
	<40	913 (42%)	1301 (50%)	59%
	40–44	313 (14%)	351 (13%)	53%
	45–49	228 (10%)	257 (10%)	53%
	50–54	530 (24%)	456 (17%)	46%
	≥55	204 (9%)	247 (9%)	55%
Road L_Aeq24h_ dB(A)				
	<40	185 (9%)	340 (13%)	62%
	40–44	318 (15%)	500 (20%)	61%
	45–49	565 (27%)	704 (28%)	55%
	50–54	658 (31%)	651 (25%)	50%
	≥55	402 (19%)	361 (14%)	47%

## 3. Results and Discussion

### 3.1. Descriptive Results

Women were more likely to respond than men (59% *vs.* 50%). Older age was associated with higher response rate and there was a negative association between road noise exposure and response rate (Table 1). Other characteristics of the study population are described in Table 2. 

**Table 2:** Socio-demographic characteristics of the responders to the 2007 Survey regarding household environment and health (total and divided by combined noise exposure).

		Total	<55 dB(A)	≥55 dB(A)
Age	Median (q1–q4)	46 (33–61)	48 (35–61)	45 (31–61)
BMI	Median (q1–q4)	24.6 (22.2–27.5)	24.6 (22.3–27.3)	24.5 (22.1–27.7)
Education	9 years or less	22% (560)	19%	24%
	High School	34% (873)	34%	35%
	University	44% (1134)	48%	42%
Place of birth	Sweden	75% (1917)	81%	68%
	Other	25% (657)	19%	32%
Strained economy	Yes	8% (215)	94%	89%
	No	92% (2340)	6%	11%
Sex	Male	46% (1192)	45%	46%
	Female	54% (1420)	55%	54%
Civil status	Co-living	67% (1723)	75%	60%
	Single/divorced	33% (835)	25%	40%
Smokers	Yes, current	25% (631)	22%	28%
	No, former/never	75% (1908)	78%	73%
Hearing impaired	Yes	19% (493)	19%	19%
	No	81% (2090)	81%	81%

### 3.2. The Benefit of Access to a Quiet Side

The proportion having access to a quiet indoor space or a window facing green space, as well as the proportion having their bedroom window facing a green space decreased with higher levels of noise from combined sources (Table 3). The benefit of having bedroom window facing green space was present at all noise levels. The overall proportion annoyed due to traffic noise, experiencing poor sleep quality or concentration problems was lower in the group having access to a quiet side, irrespective of which of the three questions that were used to assess quietness in the dwelling (Supplementary Tables S1–S3). 

**Figure 1:**
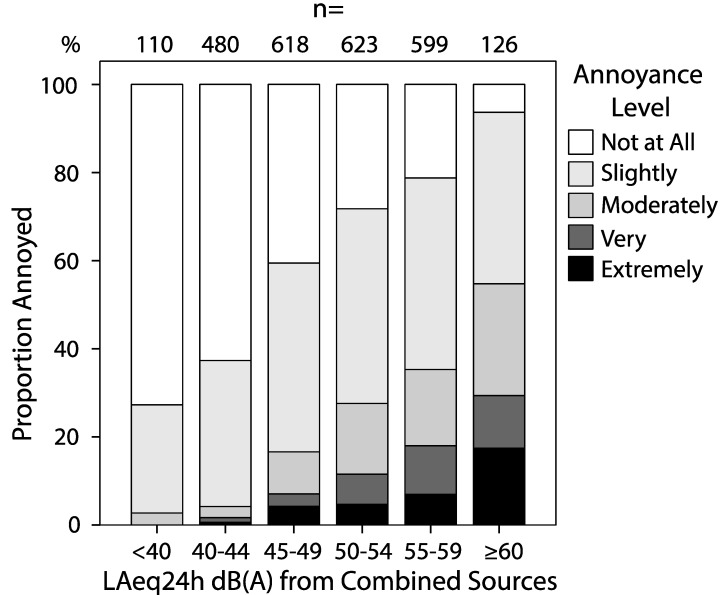
Proportion annoyed due to combined sources of noise for different noise level categories.

**Figure 2:**
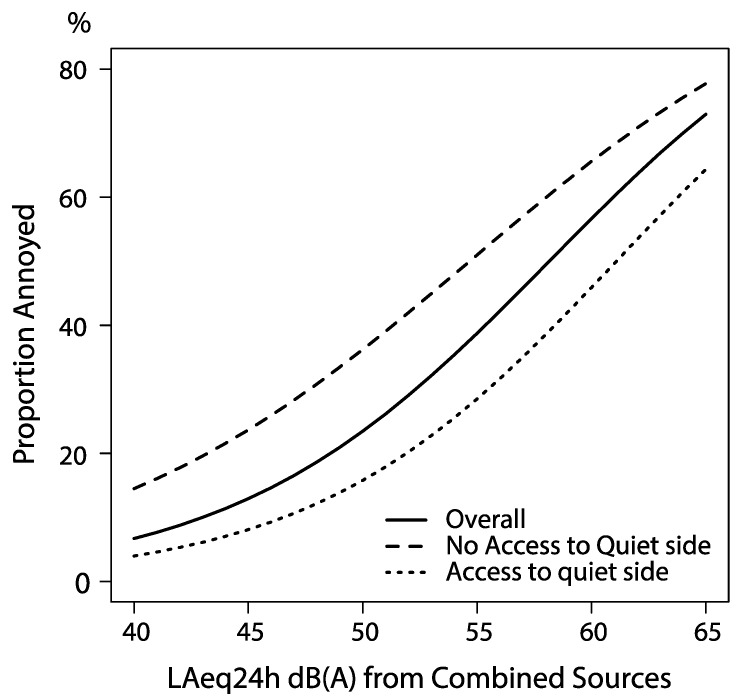
Predicted proportion of annoyed due to traffic noise and access to quiet side. Based on three separate logistic regression models (unadjusted models).

Noise annoyance from the total traffic noise was positively related to modeled noise exposure as shown in Figure 1. Window facing green space was associated with a significantly reduced risk of noise annoyance due to combined traffic noise, adjusted OR (95% CI) 0.47 (0.38–0.59) (Table 4). With access to quite side in the regression model the OR for noise annoyance decreased only marginally from 2.10 (95% CI 1.91–2.30) to 2.06 (95% CI 1.88–2.26) per 5 dB(A) increase in the noise level from combined sources. Figure 2 shows the probability of noise annoyance in relation to noise exposure. The modeled probability is based on three crude logistic regressions split by access to quiet side. The three curves have similar trajectories, but different intercepts, showing a sustained and similar beneficial effect of quiet side at all noise exposure levels. There was no significant interaction between noise level and quiet side on noise annoyance, irrespectively if noise was entered as a continuous or categorical variable (*p* = 0.87). The estimate for quiet side did not change when adjusting for other confounders stated in Table 4. Approximately 50% of those lacking quiet side (all three questions alike) were annoyed at average noise levels ranging 50–54 dB(A) while those who had windows facing a green space did not reach that proportion annoyed until ≥60 dB(A). 

**Table 3:** Access to quiet side in one’s dwelling per exposure category percent (n).

	LAeq24h dB(A) Combined						
	<40	40–44	45–49	50–54	55–59	≥60	Total
Window(s) facing green space	82% (89)	82% (392)	74% (454)	69% (429)	67% (398)	58% (72)	72% (1834)
Bedroom window facing green space	74% (80)	76% (360)	63% (386)	59% (360)	51% (302)	36% (45)	61% (1533)
Subjective access to quiet indoor space	88% (96)	84% (401)	64% (388)	55% (340)	46% (270)	37% (46)	61% (1541)
Total (n) per exposure category	110	480	618	623	599	126	2556

**Table 4:** Estimated effects of noise (un-adjusted and adjusted) and estimated effects of all included confounding factors (mutually adjusted).

	OR (95% CI)	OR (95% CI)	OR (95% CI)
	Noise Annoyance	Poor Sleep Quality	Concentration Problems
Crude			
L_Aeq24h_ Combined 5 dB(A)	2.03 (1.86–2.22)	1.26 (1.16–1.38)	1.14 (1.05–1.23)
Adjusted			
L_Aeq24h_ combined 5 dB(A)	2.10 (1.91–2.30)	1.20 (1.10–1.31)	1.09 (1.01–1.19)
Male (*vs*. Female)	0.79 (0.64–0.97)	0.75 (0.60–0.94)	0.62 (0.50–0.77)
Age	0.98 (0.98–0.99)	1.01 (1.00–1.02)	0.99 (0.98–0.99)
BMI	0.98 (0.96–1.01)	1.03 (1.00–1.05)	1.01 (0.98–1.03)
Current smoker (*vs.* former/never)	0.86 (0.68–1.10)	1.10 (0.86–1.41)	1.05 (0.82–1.33)
Single/Divorced (*vs*. co-living/married)	0.87 (0.70–1.08)	1.13 (0.89–1.42)	1.25 (1.00–1.56)
High school (*vs*. low)	1.08 (0.79–1.48)	0.96 (0.70–1.30)	0.88 (0.64–1.20)
University (*vs*. low)	1.73 (1.27–2.35)	0.85 (0.62–1.15)	1.03 (0.76–1.39)
Born outside Sweden	1.10 (0.88–1.39)	1.87 (1.48–2.37)	1.15 (0.91–1.46)
Strained economy	1.88 (1.33–2.66)	3.04 (2.18–4.25)	3.31 (2.39–4.59)
Hearing impairment	1.19 (0.90–1.56)	1.30 (0.98–1.71)	1.69 (1.30–2.19)
Also adjusted for quiet side			
L_Aeq24h_ combined 5 dB(A)	2.06 (1.88–2.26)	1.20 (1.09–1.30)	1.08 (0.99–1.17)
Window(s) facing green space	0.47 (0.38–0.59)	0.86 (0.68–1.09)	0.76 (0.61–0.95)
Bedroom facing green space	0.37 (0.30–0.45)	0.78 (0.64–1.00)	0.77 (0.63–0.96)

Overall, there was a positive relation between combined noise exposure and self-reported poor sleep quality, OR (95% CI) 1.26 (1.16–1.38) for each 5 dB(A) increase (Table 4). Having the bedroom towards green space was associated to a lower risk of poor sleep quality; 0.78 (0.64–1.00), *p* = 0.048. The benefit of having windows facing green space in relation to sleep disturbance was however not significant OR 0.86 (0.68–1.09). 

Overall, there was a positive relation between combined noise exposure and reported concentration problems, OR (95% CI) 1.14 (1.05–1.23) for each 5 dB(A) increase (Table 4). There was a clear benefit on concentration problems of generally having windows facing green space (OR 0.76; 0.61–0.95), and also more specifically having the bedroom window facing a green space (OR 0.77; 0.63–0.96). There was no significant interaction between noise exposure and quiet side, p-value for interaction >0.6 in all aspects of disturbance (noise annoyance, sleep and concentration).

Several of the included covariates were found to be strongly associated with noise annoyance, sleep quality and concentration problems (Table 4). Men were less likely to report noise annoyance, poor sleep quality and concentration problems than women. Strained economy, in this case defined as not being able to pay one’s bills on time, was associated with greater noise annoyance and the odds of reporting sleep and concentration problems was approximately three times higher in this group in the fully adjusted model (Table 4). None of the included covariates changed the estimated effects of noise exposure or access to a quite side by more than 10% in any of the three main regressions (noise annoyance, sleep, concentration).

**Figure 3:**
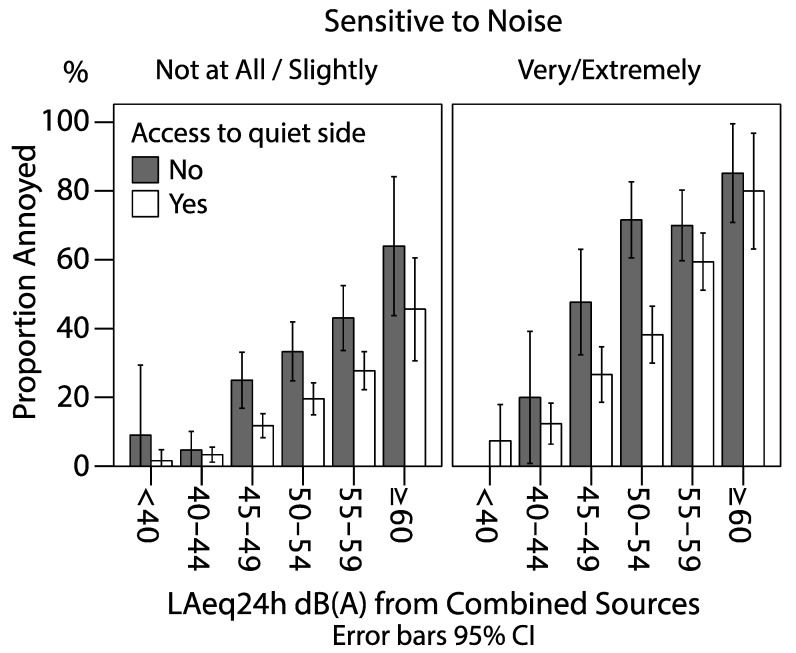
Proportion annoyed due to road traffic or railway noise, by noise sensitivity and access to quiet side at different noise level categories.

### 3.3. Noise Annoyance and Noise Sensitivity

Annoyance due to road traffic noise was affected by the responders’ noise sensitivity. In the most sensitive group consisting of *“very” and “extremely”* noise sensitive, the proportion annoyed was much higher than in the group stating not being noise sensitive at all. Odds Ratio for noise annoyance among the most sensitive was 9.2 (95% CI 6.1–14.0) compared to those not sensitive at all (1.0) in the fully adjusted model. Adding noise sensitivity to the regression model did not affect the effect estimate associated with noise exposure, and only marginally changed the estimate associated with having windows facing a green space, from OR (CI 95%) 0.47 (0.38–0.59) to 0.46 (0.36–0.57). No interaction between noise sensitivity and quiet side was discerned (*p* = 0.58). Hence, although noise sensitive persons are more annoyed to noise, they were not found to have a greater relative benefit from access to quiet side than non-sensitive individuals (Figure 3).

### 3.4. Noise Annoyance Related to Road, Rail and Combined Sources

**Figure 4:**
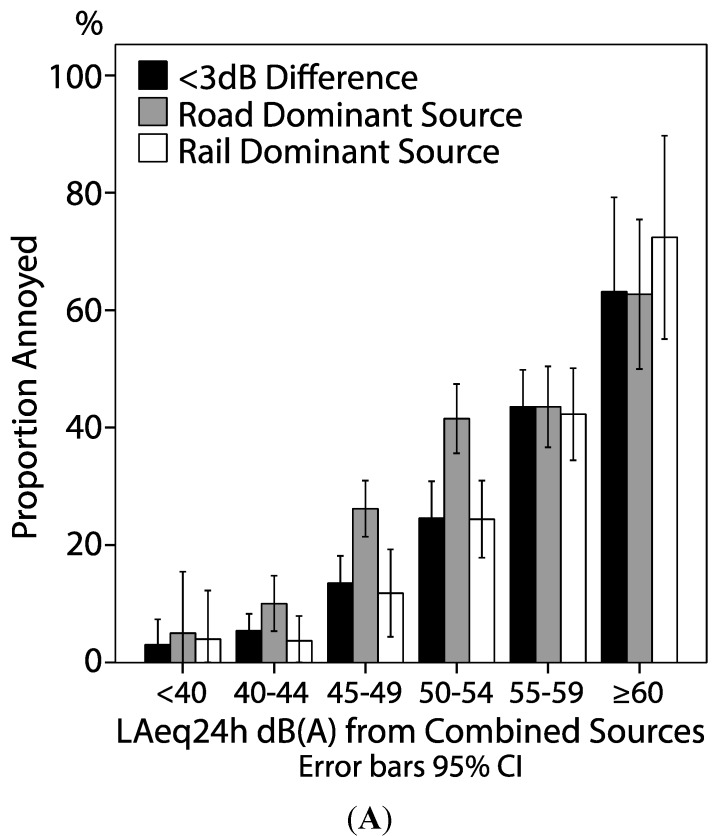

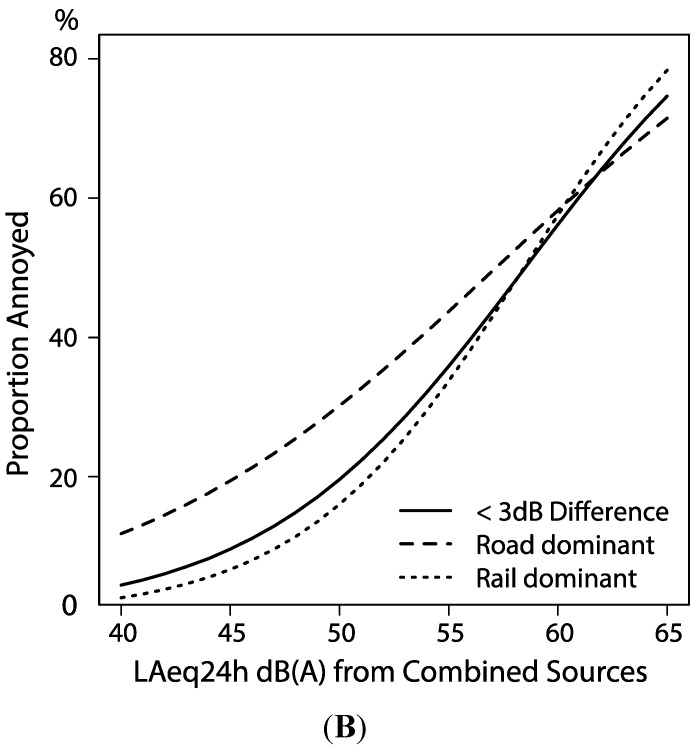
**(A**) Proportion annoyed due to road traffic or railway noise grouped by noise level and dominant noise source. (**B**) Predicted proportion of annoyed due to road traffic or railway noise, based on three separate logistic regression models (unadjusted models).

Noise annoyance when railway noise was the dominant source was significantly lower compared to equal levels of road traffic noise and noise from combined sources at noise levels 45–49 dB(A) and 50–54 dB(A) *p* < 0.01 in both comparisons. No significant difference in noise annoyance was found below 45 dB(A) or equal or above 55 dB(A) (*p* ≥ 0.1; Figure 4A). Three different crude logistic models were carried out stratified on dominant source (Figure 4B). Adjusting for median age (46) and sex (0.5) did not change the shape of the curves.

## 4. Discussion

### 4.1. Access to a Quiet Side

This study gives clear evidence of several benefits of having access to a quiet side of one’s dwelling. Having at least one window facing a yard, garden, water or green space was associated with reduced risk of noise annoyance and concentration problems. Lacking this access increased the reporting of road traffic noise annoyance (at a given exposure level at the most exposed façade) by 32%–50%. Having one’s bedroom window facing a yard, garden, water or green space was associated with reduced risk of noise annoyance, concentration problems, as well as better sleep quality. Although noise sensitive persons were more annoyed to noise, they were not found to have a greater relative benefit from access to quiet side than non-sensitive individuals when it comes to self-reported noise annoyance.

Levels of 55 dB(A) is the current WHO guideline for acceptable outdoor noise levels at the most exposed façade [28]. In the present study 32% and 43% reported being annoyed due to road traffic noise, in the 50–54 dB(A) and 55–59 dB(A) categories, respectively. For those without access to a quiet side, around half of the study population was annoyed at the same levels. 

### 4.2. Noise from Combined vs. Dominant Source

We found a clear and positive relation between traffic noise from road, railway and the two sources combined and self-reported noise annoyance, sleep and concentration problems.

Railway noise was less annoying at intermediate (45–54 dB(A)) levels compared to equal levels of road traffic noise. However, no significant difference in self-reported noise annoyance was found at other equal noise levels. This effect was consistent when adjusting for age and sex. The European union directive on community noise includes a “railway bonus” of 5 dB irrespective of noise levels, since it has been proposed to be less annoying than road traffic noise [17,29]. This study finds support for a railway bonus at low noise levels, but we cannot conclude that this bonus is justified at levels exceeding L_Aeq24h_ 55 dB(A), similar to results in a previous study [26]. Although meta-analysis show that the risk of self-reported annoyance and sleep disturbance is higher due to exposure from aircraft and road traffic noise compared to railway noise [17,29], there are new studies indicating that railway noise is at least equally disturbing when using objective measurements of sleep disturbance such as sleep medication intake [19] and polysomnography [20].

### 4.3. Strengths and Weaknesses

We used self-reported assessment of noise annoyance, concentration and sleep related problems. Many factors influence the validity of self-reporting. Several studies have found that objective measurements many times differ or in some cases are contradictory to self-reporting [30]. The survey used in this study had the explicit aim of investigating the impact of environmental factors on health. There is a risk that subjects who experience such exposures, *i.e.*, traffic noise, are more attentive to such a survey, both with regard to participation and reporting, thus resulting in both a participation and reporting bias. In one of our earlier studies comparing this survey to a broader public health survey, we did not find convincing support for context-driven bias regarding general noise annoyance. In that study sleep disturbance and concentration problems were not tested [31].

There are other important causes of poor sleep, which have to be considered. One of the most important is obstructive sleep apnoea syndrome (OSAS). Here, obesity and male sex are major risk factors, 50% of the cases are obese men [32]. Epidemiological studies have also showed a strong relation between current cigarette smoking and sleep disorders [33]. In this study we were able to adjust for both BMI and smoking, with little changes on the effect estimates of noise.

The question regarding sleep quality (“How do you usually sleep”) has been used by Öhrström *et al*. earlier, however the scale has not been validated although it is somewhat similar to the Basic Nordic Sleep Questionnaire “During the last three months, how well have you usually slept?”—“well, rather well, neither well nor badly, rather badly, badly” [34].

The question “Do you usually have difficulties concentrating on what you want to do?” with an attached 4-point scale has, to the best of our knowledge, not been validated. There are several other questions that could have been used such items from MADRS [35] GHQ-12 [36] or other. Overall, when using binominal outcomes misclassification of outcomes is likely to be non-differential with respect to road noise exposure which can bias the results towards the null. The response rate to this survey was only 54% and this may be a source of selection bias. Since we did know some of the demographic descriptive data regarding the original selection a comparison between responders and non-responders was carried out. For example response rate was lower in areas with high noise exposure levels compared to areas with lower exposure, which causes an under-estimation of the average road traffic noise exposure.

The quality of our noise exposure data has generally been detailed and of high quality based on actual measurements of traffic intensity for a majority of the road segments. We were able to combine data on vehicles for road segments belonging to the government and local municipalities. As shown in Figure 1, we observed a clear correlation between modelled exposure and self-reported noise annoyance from road traffic noise, indicating a reasonable ranking of current exposure across study subjects. Few persons were exposed to high noise levels and results regarding differential annoyance between railway and road traffic noise at levels above 60 dB(A) should be considered with care.

There are, however, some limitations of the exposure assessment. We did not have information regarding the individual buildings such as window glazing, insulation, and on which floor people lived. Nor did we consider other noise sources, e.g., noise from neighbours, air traffic, ventilation or work exposure. We also had to rely on self-reports of access to quite side, which may lead to positive bias if such reporting also reflected the actual exposure at the most exposed façade. Noise levels were connected to individual exposure via the residential building closest to the centroid of the real estate. Some study participants might therefore be assigned to the wrong residential building. This problem is expected to be largest in rural areas and areas with large buildings, the latter being more likely since the setting for this study is mainly urban. Effects on the categorical analysis should however be marginal, whereas the continuous analysis might suffer more from the precision error. 

Also, combining noise exposure from different sources has been questioned because of different acoustic characteristics for different noise sources [37]. However, while acknowledging this we believe that combining different noise sources is mainly a problem when combining road/rail with aircraft noise, both due to the very different spatial distribution patterns and differences in tonal components, acceleration *etc*. Tonal components and acceleration also differ between road and railway noise, but we and an increasing number of researchers argue that the difference in noise annoyance in relation to average noise levels from the two sources might not be all that different. Also, adding a second noise source has been shown not to increase the adverse noise reaction and it has been proposed by others that a more important violation of independence is related to variation in the spatial pattern of the exposures—*i.e.*, quiet side [38]. 

### 4.4. Results in Relation to Other Studies

This study’s results regarding the benefit of quiet side are in line with earlier studies within this field [15,16,39,40]. Öhrström *et al*. studied the impact of quiet side using modelled noise levels for both the most and least exposed façade, defining a quiet side in *absolute terms* as average noise levels <45 dB(A) [15]. They found that access to a quiet side reduced noise annoyance and other disturbances by an average of 30%–50% equal to a reduction in sound levels of L_Aeq24h_ 5 dB(A) at the most exposed side, which are very similar findings as in this study, both in absolute and relative terms. 

Three Dutch studies have investigated the effect of *relatively quiet* façade. In the first, by de Kluizenaar *et al*. in 2011 found that those living in dwellings with relatively quiet façades, defined as greater difference than 10 dB between the most and least exposed façade had a reduced risk of noise annoyance compared to those living in dwellings where the difference was less than 10 dB [40]. They also found that the effect of relatively quiet side possibly increased with higher noise levels, which is somewhat in contrast with the findings in the present study. The results may be influenced by the predominant structure of blocks, *i.e.*, “open” blocks with straight buildings along a road which are not part of a block structure sheltering from all sides, while “closed” blocks have such a sheltering effect. There is anecdotal evidence that “open blocks” are more frequent in Malmö compared to most Dutch cities, which could explain this difference. Van Renthergem and Botteldooren found that lack of access to a relatively quiet side was associated to a higher degree of noise annoyance than among those with relatively quieter sides [16]. In a recent study by de Kluizenaar *et al*. from 2013 they further investigated the effect of relatively quiet side and found roughly the same results as in the earlier studies, *i.e.*, a benefit of quiet side equaling approximately a 5 dB(A) reduction of the most exposed side if one were to lack access to a quiet side. This 5 dB shift seems to be relatively stable throughout the other studies. In our study we found a benefit of access to a quiet side corresponding to a 10 dB decrease in exposure at dwellings lacking windows facing a supposedly quiet environment and approximately 5 dB compared to the total study population. 

### 4.5. Relevance for Policy

Quiet side of dwellings have lately become a possible solution for regulators wishing to build in noisier environments. In Sweden a recent governmental report suggests that houses should be allowed to be constructed in areas exceeding L_Aeq24h_ 55 dB(A) at the most exposed façade, if at least half of the windows are faced towards a relatively quiet side [14]. A 5 dB benefit of a quiet side would in that case allow for construction in areas with up to 60 dB(A) at the most exposed façade. However, the authors to that report want to go further, claiming that modern buildings, due to improved insulation, allow for even higher noise levels. Current proposed policy changes in Sweden also rely heavily on the benefits of quiet side and that newer buildings isolate better for noise. In this context our results regarding access to quiet indoor space could be useful. We found that even with access to indoor spaces that were perceived as quiet, there was still a clear dose-response between annoyance and noise levels at the most exposed façade. Noise annoyance levels in the group with access to quiet indoor spaces were in our study shifted approximately 5 dB(A) compared to the average noise annoyance, but the noise annoyance prevalence with access to quiet indoor space was still 27% at levels 55–59 dB(A) and 41% at levels exceeding 60 dB(A). These prevalences can hardly be considered as acceptable and clearly indicates that it is not only the noise levels indoors, and with closed windows, that matter for the noise annoyance.

## 5. Conclusions

Having at least one window facing a green space was associated with substantially reduced risk of noise annoyance and concentration problems. If this window was the bedroom window, this was also true for sleeping problems. These results suggest that to protect most people (80%) from experiencing noise annoyance, the sound levels from road traffic should not exceed L_Aeq24h_ 50 dB at the most exposed façade, even if the dwelling faces a quiet side. If there is access to a perceived quiet indoor space this level could be raised to 55 dB. Although noise sensitive persons are more annoyed to noise, they were not found to have a greater relative benefit from access to quiet side than non-sensitive individuals. Railway noise was found to be less annoying than road traffic noise at lower average noise levels, but we found no support for this at levels exceeding LAeq24h 55 dB(A).

## Supplementary Files

Supplementary File 1

## References

[1] Fritschi L., Brown L., Kim R., Schwela D., Kephalopoulos S. (2011). Burden of Disease from Envionmental Noise: Quantification of Healthy Life Years Lost in Europe.

[2] (2005). Transporternas Utveckling till 2020, in Swedish.

[3] Selander J., Nilsson M.E., Bluhm G., Rosenlund M., Lindqvist M., Nise G., Pershagen G. (2009). Long-term exposure to road traffic noise and myocardial infarction. Epidemiology.

[4] Babisch W. (2006). Transportation noise and cardiovascular risk: Updated review and synthesis of epidemiological studies indicate that the evidence has increased. Noise Health.

[5] Sorensen M., Hvidberg M., Andersen Z.J., Nordsborg R.B., Lillelund K.G., Jakobsen J., Tjonneland A., Overvad K., Raaschou-Nielsen O. (2011). Road traffic noise and stroke: A prospective cohort study. Eur. Heart J..

[6] Sorensen M., Andersen Z.J., Nordsborg R.B., Becker T., Tjonneland A., Overvad K., Raaschou-Nielsen O. (2013). Long-term exposure to road traffic noise and incident diabetes: A cohort study. Environ. Health Perspect..

[7] Van Kempen E., Babisch W. (2012). The quantitative relationship between road traffic noise and hypertension: A meta-analysis. J. Hypertens..

[8] Riethmuller S., Muller-Wenk R., Knoblauch A., Schoch O.D. (2008). Monetary value of undisturbed sleep. Noise Health.

[9] Den Boer E., Schroten A. (2007). Traffic Noise Reduction in Europe.

[10] Marks A., Griefahn B. (2007). Associations between noise sensitivity and sleep, subjectively evaluated sleep quality, annoyance, and performance after exposure to nocturnal traffic noise. Noise Health.

[11] Ohrstrom E. (2000). Sleep disturbances caused by road traffic noise—Studies in laboratory and field. Noise Health.

[12] Ohrstrom E., Björkman M. (1988). Effects of noise-disturbed sleep—A laboratory study on habituation and subjective noise sensitivity. J. Sound Vib..

[13] Griefahn B., Brode P., Marks A., Basner M. (2008). Autonomic arousals related to traffic noise during sleep. Sleep.

[14] (2013). Integrated Noise Regulations to Facilitate Housing Construction (Samordnade Bullerregler för att Underlätta Bostadsbyggandet).

[15] Ohrstrom E., Skanberg A., Svensson H., Gidlof-Gunnarsson A. (2006). Effects of road traffic noise and the benefit of access to quietness. J. Sound Vib..

[16] Van Renterghem T., Botteldooren D. (2012). Focused study on the quiet side effect in dwellings highly exposed to road traffic noise. Int. J. Environ. Res. Public Health.

[17] Miedema H.M., Vos H. (2007). Associations between self-reported sleep disturbance and environmental noise based on reanalyses of pooled data from 24 studies. Behav. Sleep Med..

[18] Ohrstrom E., Barregard L., Andersson E., Skanberg A., Svensson H., Angerheim P. (2007). Annoyance due to single and combined sound exposure from railway and road traffic. J. Acoust. Soc. Am..

[19] Lercher P., Brink M., Rüdisser J., Renterghem T.V., Botteldooren D., Baulac M., Defrance J. (2010). The effects of railway noise on sleep medication intake: Results from the alpnap-study. Noise Health.

[20] Elmenhorst E.-M., Pennig S., Rolny V., Quehl J., Mueller U., Maaß H., Basner M. (2012). Examining nocturnal railway noise and aircraft noise in the field: Sleep, psychomotor performance, and annoyance. Sci. Total Environ..

[21] (2002). Directive 2002/49/EC of the european parliament and of the council of 25 June 2002 relating to the assessment and management of environmental noise. 2002/49/EC.

[22] (2007). Malmö Stad Strategisk Bullerkartläggning.

[23] Bendtsen H. (1999). The nordic prediction method for road traffic noise. Sci. Total Environ..

[24] (1996). Road traffic noise: Nordic prediction method. TemaNord, 1996:525.

[25] Naturvårdverket (1999). Buller Från Spårburen Trafik: Nordisk Beräkningsmodell.

[26] Ohrstrom E., Barregård L. (2005). Undersökning av Hälsoeffekter av Buller Från Vägtrafik, tåg och flyg i Lerums Kommun.

[27] ISO (2003). ISO/TS 15666 Technical Specification, Acoustics—Assessment of Noise Annoyance by Means of Social and Socio-Acoustic Surveys.

[28] Berglund B., Lindvall T., Schwela D.H. (2000). Guidelines for Community Noise.

[29] Miedema H.M., Oudshoorn C.G. (2001). Annoyance from transportation noise: Relationships with exposure metrics DNL and DENL and their confidence intervals. Environ. Health Perspect..

[30] Skanberg A., Ohrstrom E. (2006). Sleep disturbances from road traffic noise: A comparison between laboratory and field settings. J. Sound Vib..

[31] Bodin T., Bjork J., Ohrstrom E., Ardo J., Albin M. (2012). Survey context and question wording affects self reported annoyance due to road traffic noise: A comparison between two cross-sectional studies. Environ. Health.

[32] Young T., Palta M., Dempsey J., Skatrud J., Weber S., Badr S. (1993). The occurrence of sleep-disordered breathing among middle-aged adults. N. Engl. J. Med..

[33] Wetter D.W., Young T.B., Bidwell T.R., Badr M.S., Palta M. (1994). Smoking as a risk factor for sleep-disordered breathing. Arch. Intern. Med..

[34] Partinen M., Gislason T. (1995). Basic nordic sleep questionnaire (BNSQ): A quantitated measure of subjective sleep complaints. J. Sleep Res..

[35] Montgomery S.A., Asberg M. (1979). A new depression scale designed to be sensitive to change. Brit. J. Psychiatr..

[36] Goldberg D., Williams P. (2000). General Health Questionnaire (GHQ).

[37] Basner M., Babisch W., Davis A., Brink M., Clark C., Janssen S., Stansfeld S. (2014). Auditory and non-auditory effects of noise on health. Lancet.

[38] Miedema H.M. (2004). Relationship between exposure to multiple noise sources and noise annoyance. J. Acoust. Soc. Am..

[39] De Kluizenaar Y., Janssen S.A., Vos H., Salomons E.M., Zhou H., van den Berg F. (2013). Road traffic noise and annoyance: A quantification of the effect of quiet side exposure at dwellings. Int. J. Environ. Res. Public Health.

[40] De Kluizenaar Y., Salomons E.M., Janssen S.A., van Lenthe F.J., Vos H., Zhou H., Miedema H.M., Mackenbach J.P. (2011). Urban road traffic noise and annoyance: The effect of a quiet facade. J. Acoust. Soc. Am..

