# Supplementary material for: Annoyance, Sleep and Concentration Problems due to Combined Traffic Noise and the Benefit of Quiet Side
Source: Int J Environ Res Public Health. 2015 Jan 29;12(2):1612–28. doi: 10.3390/ijerph120201612 (PMC4344683; doi:10.3390/ijerph120201612)
Supplement: Supplementary File 1 [file ijerph-12-01612-s001.pdf]

Annoyance, Sleep and Concentration Problems due to Combined Traffic Noise and the Benefit of Quiet Side

Table S1. Proportion annoyed due to road traffic or railway noise based on objective and subjective access to quiet side percent (n).

**LAeq24h dB(A) COMBINED**		**<40**	**40–44**	**45–49**	**50–54**	**55–59**	**≥60**	**Total**
Annoyed (total)		4% (4)	7% (32)	20% (124)	32% (199)	43% (259)	65% (82)	27% (700)
Window(s) facing green space	No	5% (1)	8% (7)	32% (51)	47% (91)	54% (105)	75% (39)	42% (294)
	Yes	3% (3)	6% (25)	16% (71)	25% (108)	39% (154)	58% (42)	22% (403)
				*	*	*		*
Bedroom window facing green space	No	4% (1)	13% (15)	31% (69)	50% (126)	52% (149)	71% (57)	42% (417)
	Yes	4% (3)	5% (17)	14% (53)	19% (69)	35% (105)	56% (25)	18% (272)
			*	*	*	*		*
Subjective access to quiet indoor space	No	15% (2)	16% (12)	35% (77)	50% (140)	57% (183)	79% (62)	48% (476)
	Yes	2% (2)	5% (20)	11% (44)	17% (57)	27% (74)	41% (19)	14% (216)
			*	*	*	*	*	*

Note: * Difference between Yes and No $p < 0.05$.

Table S2. Bad sleep quality in relation to noise from combined sources, by three different measures of access to quiet side.

**LAeq24h dB(A) COMBINED**		**<40**	**40–44**	**45–49**	**50–54**	**55–59**	**≥60**	**Total**
Window(s) facing green space	no	21% (4)	16% (13)	16% (25)	24% (45)	32% (62)	24% (12)	23% (161)
	yes	9% (8)	12% (48)	18% (82)	20% (84)	22% (85)	25% (17)	18% (324)
						*		*
Bedroom window facing green space	no	14% (4)	17% (18)	19% (41)	23% (58)	29% (81)	26% (20)	23% (222)
	yes	10% (8)	12% (42)	17% (66)	20% (71)	21% (63)	23% (10)	17% (260)
						*		*
Subjective access to quiet indoor space	no	15% (2)	16% (12)	24% (53)	27% (74)	28% (90)	27% (20)	26% (251)
	yes	10% (10)	12% (49)	14% (53)	16% (55)	21% (56)	22% (10)	15% (233)
				*	*	*		*

Note: * Difference between Yes and No $p < 0.05$.

Table S3. Concentration difficulties in relation to noise from combined sources, by three different measures of access to quiet side.

**LAeq24h dB(A) COMBINED**		**<40**	**40–44**	**45–49**	**50–54**	**55–59**	**≥60**	**Total**
Window(s) facing green space	no	26% (5)	18% (15)	27% (42)	25% (47)	33% (64)	33% (16)	27% (189)
	yes	17% (15)	16% (63)	20% (91)	21% (89)	23% (89)	25% (17)	20% (364)
						*		*
Bedroom window facing green space	no	18% (5)	18% (20)	24% (52)	27% (67)	30% (85)	32% (24)	26% (253)
	yes	19% (15)	16% (56)	21% (79)	19% (69)	22% (66)	23% (10)	19% (295)
					*	*		*
Subjective access to quiet indoor space	no	15% (2)	25% (18)	29% (63)	26% (72)	32% (103)	28% (21)	29% (279)
	yes	20% (19)	15% (59)	18% (68)	19% (64)	18% (49)	28% (13)	18% (272)
				*	*	*	*	*

Note: * Difference between Yes and No $p < 0.05$.

© 2015 by the authors; licensee MDPI, Basel, Switzerland. This article is an open access article distributed under the terms and conditions of the Creative Commons Attribution license (http://creativecommons.org/licenses/by/4.0/).
